# Glycosaminoglycans and Glycomimetics in the Central Nervous System

**DOI:** 10.3390/molecules20033527

**Published:** 2015-02-19

**Authors:** Dáire Rowlands, Kazuyuki Sugahara, Jessica C. F. Kwok

**Affiliations:** 1 John van Geest Centre for Brain Repair, University of Cambridge, Forvie Site, Robinson Way, Cambridge CB2 0PY, UK; E-Mail: dr401@cam.ac.uk; 2 Proteoglycan Signaling and Therapeutics Research Group, Graduate School of Life Science, Faculty of Advanced Life Science, Hokkaido University, Sapporo 001-0021, Japan; E-Mail: k-sugar@sci.hokudai.ac.jp

**Keywords:** glycomimetics, glycosaminoglycans, central nervous system

## Abstract

With recent advances in the construction of synthetic glycans, selective targeting of the extracellular matrix (ECM) as a potential treatment for a wide range of diseases has become increasingly popular. The use of compounds that mimic the structure or bioactive function of carbohydrate structures has been termed glycomimetics. These compounds are mostly synthetic glycans or glycan-binding constructs which manipulate cellular interactions. Glycosaminoglycans (GAGs) are major components of the ECM and exist as a diverse array of differentially sulphated disaccharide units. In the central nervous system (CNS), they are expressed by both neurons and glia and are crucial for brain development and brain homeostasis. The inherent diversity of GAGs make them an essential biological tool for regulating a complex range of cellular processes such as plasticity, cell interactions and inflammation. They are also involved in the pathologies of various neurological disorders, such as glial scar formation and psychiatric illnesses. It is this diversity of functions and potential for selective interventions which makes GAGs a tempting target. In this review, we shall describe the molecular make-up of GAGs and their incorporation into the ECM of the CNS. We shall highlight the different glycomimetic strategies that are currently being used in the nervous system. Finally, we shall discuss some possible targets in neurological disorders that may be addressed using glycomimetics.

## 1. Introduction

The extracellular matrix (ECM) in the nervous system is a complex meshwork of supporting molecules arranged in diffuse, cell surface associated or tightly packed net-like formations surrounding neurons [1]. It is fundamental in maintaining the homeostasis of the central nervous system (CNS): acting as a scaffold for neuronal and glial cells, harbouring chemical signalling molecules and facilitating fast-spiking neuronal firing. As such it is a tempting target for a number of different diseases that result in neuronal disruption. Glycosaminoglycans (GAGs) are one of the major constituents in the CNS matrix [1,2]. Creating compounds that mimic the binding of glycans, either as synthetic glycans or as bioactive molecules, by competitively blocking either guidance cues or direct signalling with cells, has been termed glycomimetics. This therapeutic strategy has shown great potential in recent years with possible benefits for a range of conditions from cancer to CNS injury repair.

In this article, we shall briefly introduce the various GAGs in the nervous system, describe the unique extracellular environment of the CNS and the challenges faced by the scientists in the field of glycomimetics. We shall highlight promising research in the field of glycomimetics in both the CNS and peripheral nervous system (PNS), illustrating the different ways glycomimetic strategies can be employed to tackle different diseases. We shall conclude by providing examples of different neurodegenerative diseases which affect the extracellular matrix and which we believe may be future targets for glycomimetic interventions.

## 2. Glycosaminoglycans

Glycosaminoglycans (GAGs) are common extracellular glycans composed of linear polysaccharide chains from chondroitin sulphate (CS), heparin/heparan sulphate (HS), dermatan sulphate (DS), keratan sulphate (KS) and hyaluronan (HA) families, with CS, HS and HA being the most common members. With the exception of KS, GAGs are formed of unbranched chains of repeating disaccharide subunits composed of *N*-acetylgalactosamine (Gal*N*Ac), *N*-acetylglucosamine (Glc*N*Ac) or galactose (Gal), with glucuronic acid (GlcA) or iduronic acid (IdoA). A single GAG chain can consist of up to 50–150 disaccharide units corresponding to a molecular mass of up to 70 kDa as in the case of shark CS and CS-DS hybrid chains [3,4,5].

An integral feature of GAGs is that they can be modified with the addition of sulphate groups in a number of different positions on either sugar in the disaccharide subunit, with HA being the only exception. In the case of CS and HS, the disaccharide units are differentially sulphated on the C-4 or C-6 carbon of the Gal*N*Ac or Glc*N*Ac, on C-2, or occasionally on C-3, of the GlcA/IdoA subunit [2,6]. Sulphation for the KS disaccharide, which is composed of a Gal and Glc*NA*c subunits, occurs on the C-6 position of either Gal or Glc*NA*c or can be left unsulphated [7]. Sulphation of DS can occur on the C-2 carbon of the IdoA subunit (the presence of which distinguishes it from chondroitin sulphate) or the C-4 and/or C-6 carbons of the Glc*NA*c residues [8]. The process of GAG sulphation has been widely studied and is controlled by a number of carbohydrate sulphotransferases (Chsts) located in the endoplasmic reticulum (ER) and Golgi apparatus, such as chondroitin 4-*O*-sulphotransferases (C4STs), chondroitin 6-*O*-sulphotransferases (C6STs) and Gal*N*Ac-4-*O*-sulphate 6-*O*-sulfotransferase (Gal*N*Ac4S-6ST), thus giving a number of distinct sulphation motifs.

The levels of spatiotemporal expression of GAGs changes dramatically during development and on into adulthood [9]. The degree of glycosylation and polysaccharide chain length, together with the variation in sulphation, give rise to hundreds of potential polysaccharide structural combinations, allowing for the dynamic activation/deactivation of complex cell signalling pathways and protein binding motifs. In the CNS, different sulphation motifs are thought to govern neuronal development and adult brain homeostasis by selectively binding to axon guidance molecules and activating key cell signalling pathways [10]. In addition, the negative charge of GAGs is believed to localise extracellular ions to neurons, creating an ion reservoir which facilitates rapid neuronal firing patterns [11].

With the exception of HA, all GAGs are covalently attached to an amino acid residue on a core protein to form a proteoglycan (PG). While CS, HS and DS attach to a protein via a serine residue, KS is capable of attaching via serine, asparagine and threonine residues [7]. Chondroitin sulphate proteoglycans (CSPGs) and heparan sulphate proteoglycans (HSPGs) are the two most common PGs in the CNS and are produced by a number of cell types including both glial and neuronal cells [12,13,14,15].To date there have been at least 16 CSPGs and 15 HSPGs identified in the CNS, with some core molecules having more than one type of GAG side chain attached [15]. PGs differ in the size of the core molecule and the number of GAG side chains, with aggrecan being the most common proteoglycan and having up to 100 GAG chains consisting of around 50–150 disaccharides each [3,16]. Additionally, it is thought that core molecules like aggrecan can have different levels of glycosylation corresponding to different regions in the brain and spinal cord, further adding to the complexity of the ECM [17].

Hyaluronan is unique in the fact that it is the only un-sulphated GAG and does not attach to any core protein. It is produced in vertebrates by the transmembrane hyaluronan synthases (HAS) -1, -2 and -3 and is widely distributed throughout the body, such as in joints, connective and neural tissue [3]. Each HAS produces different lengths of the HA at different speeds, with HAS-3 producing short HA chains at slow speed and HAS-1 and -2 producing chains of up to 2000 kDa 10 times faster than HAS-3[18]. It has been recently proposed that transmembrane HAS molecules, while producing the long HA chain on the cell surface, are also acting as a docking receptors for the HA chains [19].

GAGs are known to be crucial for a range of biological functions such as cell division [20], regulation of neuronal growth and plasticity [13,14,15,16,21,22,23,24], modulation of inflammatory responses [25], cell adhesion and migration and blood brain barrier (BBB) support [20,21,22,23,24,25]. Given the importance, diversity and distribution of GAGs, it is not surprising that GAGs and their core proteins have also been implicated in a number of disease-related processes such as, tumour metastasis, toxic plaque formation and glial scar formation following injury to the CNS [13,26,27,28,29,30,31,32].

## 3. Structure and Function: GAGs in the Brain

In the CNS, GAGs are present as part of a diffuse or membrane-associated extracellular matrix or as part of a compact macromolecular structure which surround subpopulations of neurons and are termed perineuronal nets (PNNs) [1,22,23]. The formation of PNNs during early adolescence signals the closure of the “critical period”, a time during development when the brain is considered to be in its most plastic state [22]. Removing PNNs via enzymatic treatment in an adult brain reopens a critical period-like plastic state. PNN removal in targeted areas of the CNS produces profound effects such as enhancing memory retention [33] or enabling axonal regeneration following spinal cord injury (SCI) [34,35]. PNNs extend around the cell body and proximal dendrites of neurons, preferentially but not exclusively on fast-spiking GABAergic inhibitory interneurons [36]. In the CNS, they are found throughout the cortex, in a number of sub-cortical structures and nuclei and also throughout the spinal cord. PNNs help in regulating the activities of neurons in a number of ways: stabilising synaptic contacts and localising receptor turnover [37] and continually providing chemical signals that regulate the plastic potential of the neuron [38,39,40]. Because of the polyanionic properties of the PNNs, they may also act as a buffer to localise extracellular ions around the neuron facilitating the rapid firing of GABAergic neurons [11].

PNNs are highly complex macromolecular structures which contain a number of components. The transmembrane HASs produce long chains of the unsulphated HA GAGs and act as docking receptors for the PNN, anchoring them to the membrane of the neuron [19]. HA provides the backbone for the formation of the nets: while CSPGs (aggrecan, neurocan, versican, brevican) are attached via their N-terminal domain, a binding which is secured by link proteins cartilage link protein 1 (Crtl1) or brain link protein 2 (Bral2). This aggregation condenses the matrix around the neuron. The C-termini of the CSPGs will bind to the trimerictenascin-R. Up to three CSPGs can bind to one tenascin-R molecule further condensing the structure [34]. In addition to being present in PNNs, brevican and versican, along with tenascin-R also form a specialised net-like structure around nodes of Ranvier along the axon of a neuron [41].

Unlike CSPGs, which are typically located in the extracellular space, heparan sulphate proteoglycans are found both on the plasma membrane of all cells and in the ECMthroughout the body and brain [42]. In the CNS, there are 15 identified HSPGs which can be broadly classified as membrane-associated, such as syndecans or glypicans, or secreted extracellular molecules, such as perlecan and agrin [6]. This characterisation is not definitive as many HSPGs exist both as membrane attached and extracellular forms, depending on post translational modifications. They are produced by both neuronal and glial cells, and are essential in both early brain development and adult brains [43]. They interact with signalling molecules such as fibroblast growth factor and Wnts to enable neural cell proliferation [44]. Expression of the same HSPG can have local effects when expressed by a neuron or more wide spread effects when expressed by a glial cell. HSPGs like glypican 4 and glypican 6 act to establish and maintain excitatory synapses in different regions of the developing brain: glypican 4 in the hippocampus and glypican 6 in the cerebellum [45]. Pharmacological or genetic disruption of HSPGs in the hippocampus inhibits longterm potentiation, an electrophysiological response correlate to memory [46,47]. HSPGs are known to act in a range of ways with secreted factors and cell surface bound agents. They can act as co-receptors for axon guidance factors, increasing the binding between guidance molecules and their receptors as seen in the Slit/Robo signalling pathway[48].In addition, working in conjunction with CSPGs through the common ligand receptor protein tyrosine phosphatase, the binding of HSPG facilitates axon growth while the binding of CSPGs inhibits axon elongation [49]. Depending on their sulphation patterns, HSPGs can be internalised into the cell and are localised in different cellular compartments such as the nucleus, possibly aiding in the regulation of genetic expression [50,51].

## 4. Existing Strategies of Mimetics

### 4.1. Anticoagulants

One of the first successes of glycomimetics began with the identification of a pentasaccharide within heparin which facilitates its anticoagulant properties [52,53]. Heparin has been used as an antithrombotic agent in the clinic since the 1940s and is structurally very similar to HS, in that they share a common disaccharide unit [54]. The most distinguishing difference between heparin and HS in terms of expression is that heparin is exclusively expressed in mast cells while HS is ubiquitously expressed [55]. However, it was not until the 1980s that a specific pentasaccharide domain (*N*-sulphate-6-*O*-sulphate-α-d-glucosamine)1→4(β-d-glucuronic acid)1→4(*N*-sulphate-3,6-di-*O*-sulphate-α-d-glucosamine)1→4(2-*O*-sulphate-α-l-iduronic acid)1→4(*N*-sulphate-6-*O*-sulphate-d-glucosamine) which exists in some heparin chains [53] was successfully synthesised[52]. It was found that this pentasaccharide activates antithrombin III, a serine protease inhibitor, which blocks the activity of thrombin and factor Xa in the coagulation cascade. A synthetic analogue to this pentasaccharide was created and was successfully commercialised in 2002 in the US and Europe as the drug Arixtra (GlaxoSmithKline, Five Moore Drive, NC, USA). It is now widely used during post-operative care following invasive surgeries or in the sub-acute stages following stroke [56].

More recently, refinements in the development of this pentasaccharide have utilised a chemoenzymatic approach which has the potential to greatly reduce the time and cost of production. Using a series of cloned enzymes, Kuberan *et al.* (2003) were able to mimic the processing of the Golgi apparatus *in vitro* in order to create the antithrombin III-binding saccharide in sixsteps compared to the 10 steps required for chemical synthesis [57]. The authors report that the procedure produces the pentasaccharide 100 times quicker than the conventional method with the yield at least 2-fold higher. Although more work has to be performed before the chemoenzymatic approach is commercially available, this method paves a way for the future glycomimetic creation. In fact, an Arixtra-like heptamer and five HS-like oligosaccharides (two decamers, two dodecamers and one undecamer) have been chemoenzymaticallysynthesisedusing the recently improved techniques [58,59].

### 4.2. Nerve Injury Mimetics

Recovery from injury in the CNS is extremely limited. Following a CNS injury, like spinal cord injury or stroke, there is an acute glial response accompanied by cell death and inflammatory processes [60]. Astrocytes switch from a quiescent to a reactive state, moving to the site of injury and upregulating the production of inhibitory CSPGs resulting in the formation of a glial scar [61]. This CSPG-rich glial scar is inhibitory for the re-innervation of neurons and is thought to be one of the main hurdles for CNS regeneration and functional recovery [36]. As a result, severe functional deficits following traumatic injury persist into chronic stages forming lifelong debilitative conditions. In the PNS, the situation is slightly better. Long distant axonal regeneration can take place facilitating reasonable, but not complete, levels of functional recovery [62]. In an attempt to enhance recovery in both the CNS and PNS, a number of glycomimetic approaches have been recently applied in animal injury models with promising results which will be discussed below.

Polysialic acid (PSA) is a carbohydrate which is post-translationally attached to the glycoprotein—neural cell adhesion molecule (NCAM), an essential molecule for cell-cell adhesion, learning and memory, neural development and repair [63,64]. NCAM is expressed on a number of different cells in the nervous system, including neurons and glial cells. A synthetic peptide (H-NTHTDPYIYPID-OH) mimicking the PSA carbohydrate has been shown to promote motor neuron recovery following peripheral nerve injury (PNI) of the femoral nerve [65,66]. Mice treated with this glycomimetic peptide showed enhanced motor recovery following the injury. Retrograde tracing on the neurons demonstrated that the enhanced recovery was not due to improved neuronal survival nor re-innervation in the treated animals. However, the degree of remyelination on surviving axons was enhanced when compared to the saline-injected or control peptide injected animals, indicating that the mechanism is related to Schwann cell proliferation and elongation via NCAM interaction. Recently, a small compound mimetic of PSA—Tegaserod, a drug which has been approved for clinical application in an unrelated condition, has been shown to enhance locomotor recovery and sprouting of axons in mouse models of femoral nerve injury and severe SCI [67,68].

Another glycomimetic which has been used successfully in PNI is the human natural killer (HNK) cell glycan ((3'-sulfoglucuronyl beta-1,3-galactoside) epitope[66,69,70]. Like PSA, HNK-1 influences a range of functions in the nervous system including effects on synaptic plasticity, motor neuron re-innervation and learning and memory [69]. Application of the HNK-1 mimetic following PNI to the femoral nerve in mice led to enhanced functional recovery [66,70]. The HNK-1 mimetic decreased the level of neuronal deaths following injury, enhanced target re-innervation of the nerve stump and increased myelination of the nerve. The translational potential of the HNK-1 mimetic as a safe and effective therapy following PNI has been tested in non-human primates. Similar to the mouse study, adult cynomolgus monkeys (*Macaca fascicularis*) received a femoral nerve transection were treated with either a HNK-1 mimetic peptide or control peptide in silicone cuffs. Both gait and muscle force were improved in HNK-1 mimetic-treated animals of up to 160 days post-surgery. Animals in the treated group demonstrated enhanced motor reflexes. Regenerated axons were also observed to have a larger diameter. Importantly, no adverse immune effects such as antibody production or immune cell infiltration into the damaged nerve were observed after the treatment. This supports the possibility of translation of this glycomimetic from animals to human [70].

Either alone or in combination, the two glycomimetic peptides for PSA and HNK-1 have been shown to be successful in treating a PNI with a critical gap of up to 5 mm between injured segments when tethered to a collagen hydrogel [66]. Implanting a polyethylene tube filled with PSA and/or HNK-1 glycomimetic infused collagen improved functional recover and axonal re-growth across the 5 mm injury site. Because both PSA and HNK-1 glycans are attached to the extracellular matrix or cell surface *in vivo*, attaching the glycans to collagen is reflective of reality and more beneficial than infusing directly into the injury site as a suspension. This attachment not only reduces peptidase-mediated degradation of the synthetic glycans but also retention in the injected area, therefore increasing their bioavailability [66].

Interestingly, when both PSA and HNK-1 glycomimetics were tested in central rather than PNIs, the results were not as expected. Osmotic pumps were implanted subcutaneously into the backs of thoracically injured mice with a catheter providing periodic infusions of either PSA and/or HNK-1 mimetics or saline directly into the lumbar region of the spinal cord. Infusions with PSA or PSA and HNK-1, but not HNK-1 alone, immediately following injury provided the best locomotor recovery. The HNK-1 mimetic infused mice only reached scores comparable with controls, unlike the positive results observedin PNI [66,70]. The HNK-1 mimetic was seen to increase myelination of axons in the vicinity of the lesion; however, unlike the PSA mimetic, the HNK-1 mimetic failed to promote monoaminoergic innervation and enhancement of cholinergic synaptic coverage of motor neurons. If PSA mimetic infusions began 3 weeks prior to injury, the same levels of recovery could not be found, indicating that the PSA mimetic needs to be used during the acute phases of injury [71]. The potential of PSA as a therapeutic tool for SCI was confirmed in a more complete version of SCI, a thoracic dorsal hemisection which severs the ascending sensory pathways. Animals treated with PSA mimetics showed enhanced motor coordination and less reactive astrocytic gliosis which, as mentioned previously, is inhibitory to axonal regrowth [72].

Other attempts to find additional glycomimetic peptides to enhance nervous system repair have, however, been less successful. Lewis(X) is a terminal trisaccharide epitope characterised by α1,3-fucosyl-*N*-acetyl-lactosamine which is resident in the nervous system and believed to be important in neurogenesis and brain development [73]. Conversely to what was expected *in vitro*, neurite outgrowth triggered by CD24 (cluster of differentiation 24) was abolished by the Lewis(X) glycomimetic. *In vivo* assays in both peripheral and central neuroregeneration showed no benefit from the mimetic [74].

### 4.3. Stroke Injury and Hydrogels

Immediately following a cerebrovascular accident (stroke) in the brain, an injury cascade is triggered. Cells which are deprived of oxygen and glucose start to die. The result is a large cyst or cavity at the injury site, devoid of any cells or extracellular matrix [75,76]. The damage causes extensive functional impairment to an individual from which recovery is unlikely [77]. Recently, research into restoring function following stroke has focused on transplanting various stem cells, such as neural stem cells or embryonic stem cells, directly into the infarct site, to the surrounding peri-infarct region or via administration into the blood system [78]. The main problems with these approachesare that direct injection of cells into the infarct area causes the stem cells to die, and cells that have been injected into the periphery home to peripheral organs instead of the brain [79].

In order to address these obstacles, glycomimetic hydrogels have been used to promote stem cell survival when directly injected into the infarct site [80]. Hydrogels have certain benefits over more rigid scaffolds. After injection into the brain, they expand to fit to the dimensions of the cavity and are minimally invasive when compared to direct transplantation of scaffolds. Using a photothrombic stroke model in mice, a heparin-HA-collagen hydrogel containing either neural precursor cells or embryonic stem cells was transplanted into the infarct site 7 days after injury. Cells transplanted with the hydrogel were twice as likely to survive at 14 days post transplantation when compared to cells that were transplanted without a hydrogel. The gel modifies the local environment by decreasing the number of activated microglia/macrophages infiltrated to the graft site [75].

In an attempt to improve the performance of injectable glycomimetic hydrogels, the extracellular composition of different tissues has been investigated as potential hydrogel template substances [81]. Hydrogels derived from brain, spinal cord and urinary bladder matrices were tested using a three dimensional *in vitro* culture assay with a neuroblastoma N1E-115 cell line. The CNS-derived hydrogels proved to be advantageous in promoting cell differentiation and three dimensional neurite growth. There were compositional differences between the brain-derived and spinal cord-derived gels, mainly in the concentrations of sulphated GAGs and collagen. While the neurite elongation from the brain-derived hydrogel was dose dependent, the spinal cord derived hydrogel was not. This indicates that there may be a tissue specific effect on the brain derived cell line [81].

### 4.4. Blocking Viral Infections

Along with enhancing recovery following acute injury, glycomimetics have the potential to work in a number of other diseases. One of these that shows promise is in antiviral interventions. By interrupting the GAG-associated mechanisms of which a virus uses to gain access to host cells for replication, glycomimetics have the ability to stop infections from taking hold and spreading [82].

One of the main glycomimetic approaches to reducing cell infections and viral replication has been to target the cell surface dendritic cell-specific intercellular adhesion molecule-3-grabbing nonintegrin (DC-SIGN), which has been shown to bind to the human immunodeficiency virus (HIV) envelope glycoprotein gp120, facilitating docking and internalisation of the virus into host cells [83]. DC-SIGN is a C-type lectin receptor which is expressed on the surface of antigen presenting cells and macrophages. It is crucial to the early infection stages of a number of different viral pathogens like HIV, Ebola, hepatitis C or Dengue viruses [84]. Recently, a series of carbohydrate and glycomimetic DC-SIGN ligands were created with the potential of selectively blocking the function of this molecule [85]. These glycomimetics successfully compete withthe HIV-1 and Dengue viral particles in binding to the DC-SIGN on potential host cells, therefore block theinternalisation of viral particles *in vitro.*

There are also other mimetics attempted to reduce the ability of a virus to attach and internalise into a cell. A creative strategy using a combined CD4-heparan sulphate glycoconjugate has been designed to target the gp120, which normally binds to CD4 on the cell surface, and simultaneously targets other HS-binding domains required for entry that are typically hidden. The glycoconjugate, mCD4-HS_12_, was able to inhibit HIV-1 infiltration in peripheral mononuclear cells [86].As well as being used to intervene with the cycle of HIV and Dengue viruses, glycomimetics have also been tried in interrupting influenza viruses. γ-polyglutamic acid-based glycopeptides (γ-PGA) with differing α2, 3/6 sialylatedglycans have been studied in influenza virus. γ-PGAglycopeptides with short glycans were found to be effective in inhibiting avian influenza virus while γ-PGA glycopeptides with long glycans were effective in inhibiting human influenza virus *in vitro*[87].

### 4.5. Hyaluronan as a Drug Delivery System

Another way in which GAGs can be used in disease treatments is to harness the native characteristics of GAGs in order to deliver desired molecules such as drugs. Hyaluronan, being biodegradable, non-toxic, non-immunogenic and hydrophilic, is uniquely suited for this role.

Hyaluronan is a popular vehicle for drug delivery and gene regulation in cancer research. The primary reason which makes HA an ideal choice for cancer intervention is that two HA receptors, the hyaluronan receptor CD44 and the receptor for hyaluronic acid-mediated motility (RHAMM or CD168), are strongly implicated in cancer cell signaling[88,89].In addition, HA interacts with intracellular adhesion molecule-1 (ICAM-1), toll-like receptor-4 (TLR-4), hyaluronan receptor for endocytosis (HARE), and lymphatic vessel endocytic receptor-1 (LYVE-1) [90,91,92,93]. CD44, which is a cell adhesion molecule (CAM), is the most studied HA receptor in cancer research and is commonly found on the cell surface of epithelial, hematopoietic and neuronal cells. It is over-expressed in many cancer types [88,94]. The CD44 receptor controls the internalisation and metabolism of HA and requires activation for its function. HA has several chemical groups on which therapeutic drug molecules can be conjugated. The carboxyl group on the GlcA, the hydroxyl group on the *N*-acetylglucosamine and the reducing terminus are sites where modifications can be made. In addition, the acetyl group can be removed to create another site for drug conjugation. A number of anticancer drugs for a range of different cancer types have been successfully conjugated to HA chains, with HA plus irinotecan reaching phase III trials for colorectal cancer [95].

Along with drugs, it is also possible to conjugate other non-organic materials for therapeutic and diagnostic purposes. Boron atoms, which are used for boron neutron capture therapy for a number of different cancers, have been designed to bind to HA via an ester linkage. Using two different constructions, boron atoms were selectively internalised into a number of cancer cell lines [96]. For a complete review on the uses of HA for the diagnosis and treatment of cancer, refer to the review by Arpicco *et al.* [97].

## 5. Mimetics and Neurodegeneration: Possible Targets and Future Strategies

Although the field of glycomimetics is becoming well established, to date there is still a lack of research into possible glycomimetic interventions for complex neurological and neuropsychiatric disorders. This is surprising given the growing evidence that dysfunctions in the ECM play a significant role in the pathogenesis of these complex disorders. Here, we introduce a handful of these disorders and speculate how they can be served as targets of future glycomimetic interventions.

### 5.1. Schizophrenia

Schizophrenia is a chronic disorder characterised by abnormal psychotic symptoms which impair the ability of an individual to function. Symptoms often first manifest at around the age of 20 and can be broadly categorised into either positive or negative symptoms. Positive symptoms include sensory hallucinations and delusions while negative symptoms include social withdrawal and apathy. Although the causes of schizophrenia are still unknown, it is believed that a combination of genetic and environmental factors play a role in its development.

There is a growing amount of evidence linking matrix abnormalities with schizophrenia [98,99,100,101]. The majority of this has come from histological and genetic analysis using post-mortem brains of individuals who suffered from psychotic symptoms. Alterations in the number of PNN-positive neurons have been observed in a number of brain regions. Individuals suffering from schizophrenia showed a 70%–76% decrease in PNNs in the prefrontal cortex, the brain region which is responsible for decision making, planning complex action, personality, and the regulation of social behaviour [98]. This discrepancy was not observed in their visual cortex, another region full of PNNs. Interestingly, this deficit was also not observed in individuals with bipolar disorder, a related mental disorder. The difference between the extracellular environment of schizophrenic and bipolar brains also extends to the amygdala (memory, decision making, emotional reactions) [99] and the entorhinal cortex (declarative memories and spatial memories) [100], highlighting a potential difference in the pathology between these similar disorders. This histological evidence has been confirmed with RNA analysis. The RNA expression of layer 3 cortical pyramidal neurons was examined. The results suggest that the expression of proteins that are key to PNN formation and stabilisation, such as HAPLN1, aggrecan and versican, is downregulated in schizophrenic brains, while the expression of proteases that are known to digest on ECM such as ADAMTs 1 and 6, and MMP-24 is increased [101].

The primary cause of the disruption to the extracellular environment in schizophrenia is currently unknown. To date, disturbances in both PNNs and PNN-enwrapped neurons have been identified in schizophrenia in a number of brain regions including the multiple layers of the cortex and amygdala [98,101,102]. Whether it is the dysfunction of the cells that causes a PNN deficit in schizophrenic brains or whether the malformation in the PNNs causes these cell types to be more susceptible to processes like oxidative stress is unknown. It is more likely that one causes the other leading to a cascade failure. Environment plays a crucial role inthe development of schizophrenia, it is likely that deficits in this network cause behavioural problems resulting in social isolation and further exasperating epigenetic factors, creating a behavioural feedback loop.

There are a number of potential glycomimetic targets for this disorder. Two single nucleotide polymorphisms (SNPs) affecting the *STX* gene which encodes for polysialyltransferase STX (which synthesises the polysialic acid glycan) have been reported in a genome-wide association study of schizophrenic individual: SNP7 and SNP9, yielding two silent mutations [103]. In the brain, NCAM-bound PSA was found to have a dopamine-binding affinity which was lost following SNP7 mutation. In addition, PSA is known to interact with neurotrophic factors such as brain-derived neurotrophic factor. PSA mimetics that have been used in CNS and PNS injury could have the potential to amelioriate this disorder in individuals with these specific genetic mutations [66,70,71,72].

An additional mimetic strategy may be to target a chemorepulsive axon guidance molecule, semaphorin 3a (Sema3a), which has been found to be increased in the cerebellum of schizophrenic patients [104]. Sema3a has been recently found to be associated with PNNs in the adult brain [39], binding specifically to the chondroitin 4,6-sulphate (or CS-E) glycan[40], and this binding is blocked by an CS-E-binding antibody GD3G7 specifically *in vitro*[23,105].

### 5.2. Neurodegenerative Diseases

Neurodegenerative disease like Alzheimer's disease and Parkinson’s disease are characterised by progressive loss of neurons, resulting in agradual cognitive and/or motor decline. These conditions lead to a drastic reduction in the quality of life of patients. Although the exact mechanisms for cell death are unknown, these neurodegenerative diseases have characteristic neuropathologies, such as amyloidopathies that have been shown to be toxic to cells [106]. Pathologies like these are known to have relationships with ECM molecules and are, therefore, open to glycomimetic interventions.

HSPGs, together with CSPGs in a lesser extent, have been associated with extracellular β-amyloid (Aβ) senile plaques in the brain parenchyma and amyloid deposits around blood vessels, which are characteristic pathological hallmarks of Alzheimer's disease (AD). Aβ has been shown to be toxic to neurite growth *in vitro* [106]. Aβ plaques are known to have a defined zonal organisation, composed of a core zone which has a complete loss of cellular structures and the more peripheral coronal zone. The peripheral zone is characterised by damage and loss of neurons, dystrophic neurites and reactive astrocytes [107]. No changes in ECM molecules, such as aggrecan, link protein and tenascin-R, could be detected in this region with the exception of hyaluronan, which showed an increase of 26% in the temporal lobe of AD patients [108]. This increase could be a result of reactive astrocytes interacting with hyaluronan via their CD44 receptors in the vicinity of the plaque [109]. PNNs have been found to be protective of neurons, possibly from harmful reactive oxygen species [36,110].

In disease pathology where there is a progressive decline in the number of neuronal cells like that found in AD, it may be necessary to encourage replacement of cells or re-establishment of neuronal connections. By targeting the nerve growth factor (NGF)/neurotrophic tyrosine kinase receptor type 1 (TrkA) signalling pathway with a number of different CS-E constructs, it is possible to trigger neuro-differentiation and neurite outgrowth in PC12 cells *in vitro* [111]. Complementary to this, these CS-E constructs may also be useful in reopening plasticity in neurons. Orthodenticlehomeobox 2 (Otx2) has been found to bind to the PNNs of GABAergic parvalbumin (PV)-expressing interneurons by means of a 15 amino acid peptide domain containing an arginine-lysine doublet (RK peptide) within Otx2 [38]. This binding appears to be mediated by CS-D and CS-E motifs. Direct infusion of the RK peptide competitively binds to the PNNs, hence reducing the amount of PNN-bound Otx2. This causes a reduction in PNN and PV immunoreactivity and reopens plasticity in mice allowing new connections to be made [38].

### 5.3. HIV-Associated Neurocognitive Disorders

Acquired immunodeficiency syndrome (AIDS) has a potential risk of leading to neurological disorder termed HIV-associated neurocognitive disorders (HAND). In spite of antiviral treatment, approximately 40%–50% of people infected with HIV are believed to suffer from HAND [112]. HIV-associated dementia, the most severe form of HAND, affects 2%–8% of HIV sufferers despite of antiviral treatment. In the brain, the HIV virus preferentially infects microglial cells, which then go on to release ECM degrading factors such as matrix metalloproteinases (MMPs) [113]. Levels of MMP 2, 7 and 9 are elevated in the cerebrospinal fluid of patients with HAND compared to controls. This observation has been confirmed and verified *in vitro*. Cells infected with the virus also produce elevated levels of MMP2, 7 and 9 [114]. This activity has the potential to reduce the numbers of PNNs in the cortex. HIV infected brains seem to have higher PNN degradation in layers V to VII of the cortex than layers II and III [115]. It is believed that this kind of degeneration can cause the dementia-like symptoms experienced in some HIV patients. Given the success of targeting DC-SIGN in peripheral immune cells, it is interesting to speculate if a similar strategy could be used in the CNS to minimise activation of microglia and therefore the production of MMPs. Although microglia only seem to express DC-SIGN in low levels after induction via IL-4 and GM-CNS and LPS (approximately 100-fold lower than in monocytes), they do express a number of HSPGs that are thought to facilitate viral infection. The HSPGs syndecan-1 and perlecan have been found to be essential for initial docking of the virus to monocyte-derived macrophages. Treating cells with heparitinase which digests HS, inhibits HIV-1 binding to cells and lowers viral infection. Considering the unique immunological and extracellular environment of the CNS, different glycomimetic strategies are likely to be required when targeting viruses that can affect both the CNS and PNS.

### 5.4. Oxidative Stress

An imbalance between the presence of reactive oxygen species (ROS) and the ability of the body to mitigate or repair the damage is known as oxidative stress, and is thought to play a key role in a number of different neurological disorders. Alzheimer’s disease, Parkinson's disease, traumatic injury and schizophrenia are all thought to have an element of oxidative stress in their pathologies. Emerging evidence has pointed to a neuroprotective role of PNNs against ROS due to its polyanionic property. Neurons positive for *Wisteria floribunda agglutinin* (WFA), a lectin which binds in high affinity to the component in the PNNs, have been shown to be more resistant to iron-induced neuronal cell death compared to neurons that lack PNNs [116]. In humans, cells with thinner PNNs, such as cortical pyramidal neurons, appear to be more susceptible to iron oxidative stress. Neurons with PNNs also have higher loads of inter lysosomal lipofuscin [117]. Lipofuscin is formed through ion-catalyzed oxidation, and is thought to be a characteristic indicator of neurofibrillary degeneration. This has been found in both control and AD brains [36]. Intact and mature WFA-labelled PNNs are spared from degeneration in mice with a genetically induced redox imbalance compared to cells lacking WFA-nets [118]. This protective quality is thought to decrease lipid peroxidation, DNA damage and protein oxidation. Excessive oxidative stress has been shown to damage WFA-labelled nets, breaking down both HA and CS GAGs [116].Considering all the evidence pointing towards a neuroprotective role of PNNs on neurons, it might be beneficial to protect PNNs from degradation either by designing mimetics that block the activity of matrix degrading factors, such as MMPs, or by overexpressing GAGs and GAG production. This would be particularly beneficial in neurological disorders like HIV-dementia, schizophrenia and epilepsy where PNNs are known to be degraded.

### 5.5. Differentially Affected GAGs/PGs in Different Diseases: Other Targets

The number of complex neurological disorders involving ECM dysregulation has been growing and may be suitable targets for glycomimetic intervention. For example disturbances in HSPG expression has been linked with autism spectrum disorders. Studies have identified autism-like social and communication abnormalities in mice lacking HS [119]. Recently, post-mortem data from brain tissue of autistic patients has identified a lack of HS in the subventricular zone, the site of neurogenesis in the adult brain [120]. A more complete list of neurological diseases and associated type of ECM glycan deficits is found in Table 1.

**Table 1:** A list of neurological diseases and their associated ECM deficits.

Disease Type	ECM Involved	Changes	Reference
Schizophrenia	PNNs HAPLN1 Aggrecan Versican	Decrease Decrease Decrease Decrease	[98,101,118,121,122]
Stroke	CSPGs Hyaluronan	Increase Increase	[123,124]
Spinal Cord Injury	CSPGs Neurocan Brevican Phosphocan	Increase Increase Increase Increase	[13]
Autism	HSPGs	Decrease	[119,120,125]
Amyloidopathies	Hyaluronan HSPGs	Increase Increase	[27,28,29,126]
Prion’s Disease	CSPGs and PNNs	Decrease	[127]
Epilepsy	Neurocan(full length) Phosphacan Brevican	Increase Decrease Cleaved	[128,129]
Multiple Sclerosis	Hyaluronan Aggrecan Versican Neurocan	Increase Decrease Decrease Decrease	[130,131]
Human Immunodeficiency Virus Dementia	PNNs	Decrease	[113,115]

## 6. Conclusions

GAGs have proven to be exciting targets for intervention due to the wide range of biological functions with which they are associated in the body and brain. Their complexity and diversity provide a large number of potential glycomimetic strategies which could be employed as treatments for a range of disorders. Recent advances in the production of synthetic GAGs have enabled scientists to be extremely creative in their approach to disease.

Many different glycomimetics are already showing promise [63,65,66,70,71,72,75,85]. PSA and HNK-1 mimetics have been used successfully to promote axon growth, even over long distances, following axonal injury in both the PNS and CNS. HA hydrogels have been applied to stroke research with positive results for stem cell graft survival. The ability to inject a scaffold directly into the brain that conforms to the dimensions of the lesion site is a great step forward in creating an environment facilitating the re-seeding of stem cells. Several studies have demonstrated the possibilities in blocking viral transmission using a number of custom glycomimetics in different viral models. Although most of these works have yet to be studied in detail in *in vivo* trials, these findings show great promises in the future treatment of serious viral infections.

The field is still wide open to explore how glycomimetics could play a major role in the treatment of neurological disorders, such as schizophrenia and Alzheimer’s disease. Although the exact pathology of many of these diseases still remains a mystery, our understanding about processes of inflammatory responses and neuronal cell death, offers a range of potential targets for intervention. Glycomimetic strategies that aim to reduce inflammation, encourage cell proliferation and enhance synaptic plasticity could go a long way in alleviating symptoms of these debilitating diseases.
